# A Prognostic Model of Angiogenesis and Neutrophil Extracellular Traps Related Genes Manipulating Tumor Microenvironment in Colon Cancer

**DOI:** 10.7150/jca.85778

**Published:** 2023-07-09

**Authors:** Dongsheng Zhang, Yan Zhao, Shirui Wang, Xiaowei Wang, Yueming Sun

**Affiliations:** 1 Department of Colorectal Surgery, the First Affiliated Hospital of Nanjing Medical University, Nanjing, China.; 2 Colorectal Institute of Nanjing Medical University, Nanjing, China.; 3 The First School of Clinical Medicine, Nanjing Medical University, Nanjing, China.

**Keywords:** Colon adenocarcinoma, Angiogenesis, Neutrophil extracellular traps (NETs), Tumor microenvironment (TME), Prognosis

## Abstract

Colon adenocarcinoma (COAD) is one of the most common carcinomas worldwide. The main causes of cancer-related mortality of COAD are metastases. The fundamental processes for how angiogenesis and neutrophil extracellular traps (NETs) contributing to tumor progression and metastasis are still uncertain. In our study, The Cancer Genome Atlas (TCGA)-COAD dataset (train set) and GSE17536 (test set) were analyzed. Angiogenesis potential index (API) and NETs potential index (NPI) based on angiogenesis and NETs-related genes were respectively built using bioinformatic methods and machine learning algorithms. Subjects were split into groups with low API/NPI or high API/NPI. Survival analysis showed the high API and high NPI patients with the worst survival compared with the others. Between the high API/NPI group and the other groups, differentially expressed genes (DEGs) were found. A four-gene signature (TIMP1, FSL3, CALB2, and FABP4) was included in a risk model based on least absolute shrinkage and selection operator (LASSO) analysis. Additionally, the model displayed a significant association with many immune microenvironment characteristics. Finally, we verified the clinical significance of CALB2 expression and its role to promote the invasion and migration of colon cancer cells in vitro.

## Introduction

Colon adenocarcinoma (COAD) is one of the most common carcinomas worldwide. The most significant prognostic variables for poor outcomes in COAD patients are invasion and metastases^1^. Because of the lack of identifiable early indications and the absence of an efficient early diagnostic method, over half of COAD patients have an advanced stage at diagnosis. Nowadays, great progress has been made against COAD. Apart from surgery and traditional chemotherapies, novel treatment strategies are available for COAD patients with advanced stages, including targeted therapy and immunotherapy^2^,^3^. Unfortunately, side effects and drug resistance emerge, which impedes recovery. New prognostic signatures and potential therapeutic targets for the treatment of COAD may be discovered by research of important molecules and mechanisms contributing to cancer progression and metastasis.

Angiogenesis is an important mechanism for initiating the invasion and metastasis of malignant tumors. Angiogenesis in tumor tissues provides sufficient nutrients and oxygen to the infinitely proliferating tumor cells, which is an important fundament for tumor proliferation and metastasis^4^. In the presence of metabolic stress, immune/inflammatory response, and genetic mutations, some tumors acquire the ability to induce and maintain angiogenesis, which is referred to as the "angiogenic switch"^5^,^6^. It is generally accepted that angiogenic vessels formed in primary tumors are structurally abnormal and functionally poor^7^. Nevertheless, the nutrition required for tumor growth and the provision of conduits for metastatic spread of the escaping tumor cells depends on this seemingly compromised vascular system^8^. Therefore, abnormal tumor vascular architecture may mark the metastasis of tumor cells 9. Angiogenesis is regulated by multiple pathways, such as FGF, Notch, angiopoietin, VEGF, PDGF, and HGF signaling^10^. Blocking neovascularization has the potential to inhibit tumor progression, and "anti-angiogenesis" has been applied in cancer treatment.

In 2004, the discovery of a fascinating phenomenon of neutrophils caught great attention from academia. The structure known as neutrophil extracellular traps (NETs) is generated when neutrophils recondense their lobulated nucleus and release chromatin into the extracellular environment in response to specific stimuli^11^. The main defense function of NETs was thought to be the capture and eradication of bacteria and other pathogens, a variety of other pathological processes, including immunodeficiency, autoimmunity, diabetes, atherosclerosis, cystic fibrosis, and cancer, were discovered to have close relationships with NETs^12^. NETs have recently been found to play significant roles in the development of tumors, angiogenesis, metastasis, and tumor-associated thrombosis^13^-^16^. Besides, tumors can prime neutrophils to form NETs in the absence of infection or external stress to promote metastatic progression^17^. The NETs score was associated with a poor survival in patients with the major types of cancer, according to a recent study that developed a marker for pan-cancer prognosis centered on NETs^18^. A combined analysis of NETs with other metastasis-related phenotypes may lead to new discoveries.

**Figure 1:**
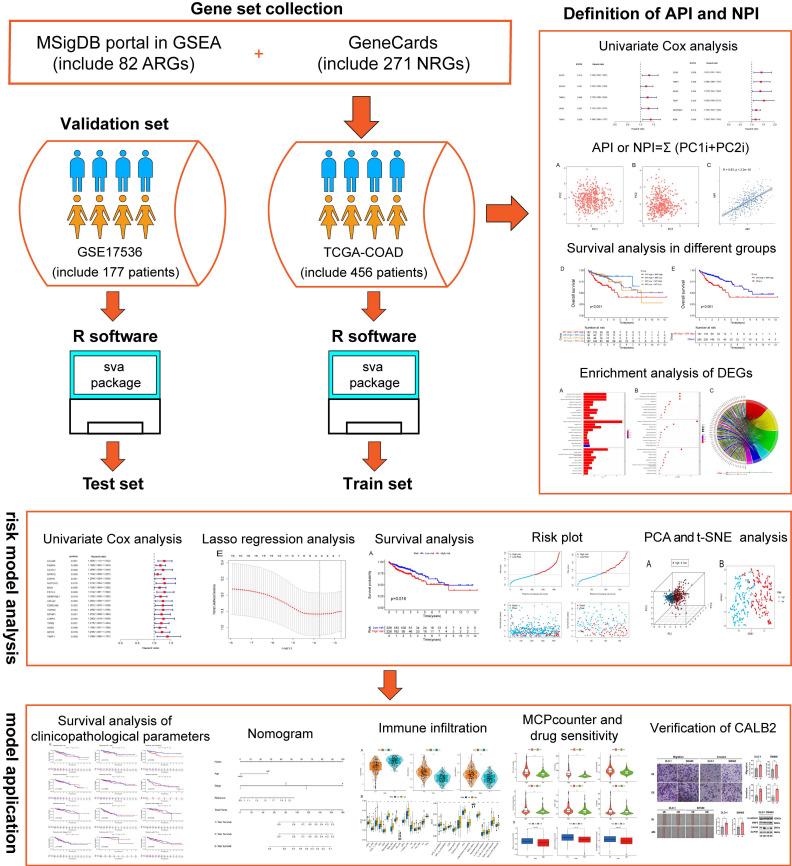
The flowchart of overall study methods and results.

To estimate clinical practice and describe the difference in survival, it is crucial to discover prognostic indicators linked with invasion and metastasis. In this study, key prognostic genes and a prognostic model were found to provide an understanding of the molecular mechanisms behind the impacts of angiogenesis and NETs in COAD. A stepwise approach was applied. First, utilizing TCGA COAD dataset, angiogenesis and NETs-related DEGs between COAD and normal tissues were identified. Prognostic genes were identified among these DEGs. Second, these prognostic DEGs were utilized to establish the API and NPI, respectively. Thirdly, DEGs between the high API /NPI and the other groups were screened. Then a prognostic model was created through LASSO regression analysis. Lastly, enriched pathways, immune microenvironment, drug sensitivity as well as tumor mutational burden (TMB) were investigated. In addition, we chose CALB2 as a target for experimental validation and discovered that it might influence colorectal cancer (CRC) cell invasion and migration in vitro. The flowchart was displayed (Fig. 1).

## Materials and Methods

### Gene Expression Data Acquisition and Processing

The RNA-seq transcriptome data, somatic mutation information, copy number variation (CNV), and clinicopathological information for COAD patients were downloaded from TCGA and the Gene Expression Omnibus (GEO) database (GSE17536)^19^. Patients with no other malignant tumors, within the age of 18 - 80 years old, who had not received chemotherapy and/or radiotherapy before surgery were enrolled. Patients with incomplete clinicopathological data were excluded. The Sva Package was applied to remove batch effects (version 3.42.0)^20^.

### Establishment of API and NPI

82 genes associated with angiogenesis were selected from the Molecular Signatures Database v7.0 (MSigDB, www.gsea-msigdb.org), 271 NETs-related genes were obtained through the GeneCards (https://www.genecards.org) (Supplementary Table 1). The limma package was employed to examine the DEGs between COAD and normal colon tissues^21^. |Log2(Fold Change (FC)) | >0.848, *P* < 0.05 were set as the cutoffs. By applying univariate Cox regression analysis of the "survival" tool, prognostic DEGs were identified. Principal component analysis (PCA) was applied with these prognostic DEGs. Like previous studies^22^, the API and NPI were separately defined: API or NPI = ∑(PC1i+PC2i), where i represented the expression of angiogenesis and NETs-related genes.

### Identification of Key high API/NPI Related Genes

Subjects were split into low or high API/NPI groups. DEGs between the high API/NPI group and the other three groups were examined through the limma package. |Log2FC| > 0.8 and *P*<0.05 were used. Gene Ontology (GO) and Kyoto Encyclopedia of Genes and Genomes (KEGG) enrichment analysis of DEGs were carried out utilizing the "clusterProfiler" software^23^.

### Development of API/NPI-related Prognostic Model

Prognostic API/NPI-related genes were identified with univariate Cox regression analysis (*P*<0.05). The glmnet package (version 4.1-3) was applied to incorporate the best candidate genes into the LASSO regression approach, which was then employed to create the API/NPI-related prognostic model^24^. The LASSO regression method was applied to establish the optimum penalty parameters through 1000 times of cross-validation, and the correlation coefficient criterion was based on the minimum criterion. The expression and coefficient of putative API/NPI-related genes were utilized to determine each subject's risk score. The formula was as follows: risk score = 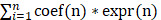, where coef(n) represented the coefficient value and expr(n) represented the gene expression. Afterward, the subjects were grouped into high- and low-risk groups depending on the median value. To assess the predicted sensitivity of the formula, Receiver Operating Characteristic (ROC) curves were presented utilizing the "survivalROC" package. Using the same coefficients and cutoff settings as the train set, the model's performance was assessed on the test set. Using the "Rtsne" and "ggplot2" tools, the subjects were presented in two dimensions via PCA and t-distributed stochastic neighbor embedding (t-SNE) techniques. The univariate and multivariate Cox regression analyses were done to find the independent predicting variables through "survival" packages. A nomogram was formed by "rms" tools to evaluate the probability that COAD patients would survive.

### Assessing the Immunological Profile of the Tumor Microenvironment (TME)

MCPcounter algorithms and Timer 2.0 (cistrome.shinyapps.io/timer/) was capable of identifying immune cell infiltration^25^. According to distinctive characteristics of the transcriptional profiles, the Estimation of STromal and Immune cells in MAlignant Tumours using Expression data (ESTIMATE) might well be able to infer the cell composition and tumor purity. The single-sample gene set of the enrichment analysis (ssGSEA) was processed by the "gsva" tools to detect the activity state of 16 immune cells and 13 immunoregulatory functions. Depending on the findings of a recent analysis, immune-checkpoint genes were chosen^26^. In addition, each sample's expression of immune checkpoints was measured, as well as that of human leukocyte antigen (HLA) genes.

### Immunohistochemistry (IHC)

80 paired CRC and adjacent normal tissues were collected from CRC patients in our hospital. The Ethics and Research Committees of Nanjing Medical University authorized this work. All patients provided informed consent after being properly informed about the research. The patients included in our study met the following criteria: (1) patients who received radical surgical resection in our hospital; (2) patients older than 18 years old;(3) patients who had received no chemotherapy and/or radiotherapy before surgery; (4) patients with complete clinicopathological data. The patients with multiple primary CRC or with a history of any other malignancy were excluded. IHC was performed as previously described^27^. The IHC staining was inspected by an experienced pathologist and analyzed with Aipathwell Software (Servicebio, Wuhan, China). H-SCORE was used to quantify the expression level of CALB2 (Supplementary Table 2). H-SCORE was calculated as follows: H-SCORE=∑ (pi×i) = (percentage of weak intensity ×1) + (percentage of moderate intensity ×2) + (percentage of strong intensity ×3)^28^,^29^.

### Cell Culture and Transfection

Human CRC cell lines (DLD-1 and SW480) were obtained from the Cell Center of Shanghai Institutes for Biological Sciences. Cells were cultured in Dulbecco modified Eagle medium supplemented with 10% fetal bovine serum, 100 U/ ml penicillin, and 100 μg/ml streptomycin at 37 °C with 5% CO2. The CALB2 overexpression plasmid was applied by genomeditech (Shanghai, China). Cell transfection was performed by Lipofectamine™ 3000 Transfection Reagent (Thermo Fisher Scientific, US) as instructed by the manufacturer.

### Transwell and Wound-healing Assay

The transwell migration assay was conducted with Millipore cell culture inserts (24-well insert, 8 μm pore size). 2*10^4^ cells were plated with 200 μL of serum-free medium. The well below was filled with 800 μL of 10% serum-containing culture medium. After 48 hours, migratory cells were stained with crystal violet solution while cells remained on the polycarbonate membrane were removed with swabs. Cells in each well were counted by three random views under a microscope. For the transwell invasion experiment, Matrigel (BD Biosciences, US) was coated onto the membrane, and the other steps were performed as described above.

For the wound-healing assay, 1*10^6^ cells were seeded into each well in 6-well plates, and after the formation of a confluent cell monolayer, a gap was made using a 200-μL pipette tip. Then, serum-free media were used to cultivate the cells. Under a 10x microscope, pictures were taken at the beginning and after 48 hours. The diminishing area was measured and normalized to the area at 0 h.

### Western Blotting (WB) Assay

Cells were lysed by Radio Immunoprecipitation Assay (RIPA) Lysis buffer (Beyotime, Shanghai, China), and the quantity of protein was determined using a bicinchoninic acid (BCA) Protein Assay Kit (Beyotime, Shanghai, China). Extracted proteins were loaded, separated by SDS-PAGE, and blotted onto PVDF membranes (Millipore, MA, USA). Membranes were then blocked using 5% non-fat dry milk and incubated with primary antibody at 4°C overnight. The following primary antibodies were used: anti-E-cadherin (1:2000; #60335-1-Ig, Proteintech, Wuhan, China), anti-CALB2 (1:2000; #66496-1-Ig, Proteintech, Wuhan, China), anti-MMP9 (1:2000; #10375-2-AP, Proteintech, Wuhan, China), and anti-GAPDH (1:5000; #5174, Cell Signaling Technology, MA, USA). The membranes were then exposed to the secondary antibody. Finally, using the Omni-ECL chemiluminescent reagent (#SQ201, epizyme, Shanghai, China), protein blots were identified with the Tanon 5200 Multi Chemiluminescent Imaging System (Tanon, Shanghai, China).

### Statistical Analysis

R V.4.1.3 and its relevant packages were utilized for statistical methods. Zero-mean normalization was applied to standardize the train and test sets. We used the Kaplan-Meier (KM) analysis to evaluate the statistical significance of differential groups through log-rank test. Nomogram construction and validation made it possible to visualize and perceive the multivariable Cox regression analysis by incorporating multi-forecast indicators. The Wilcoxon test was performed to identify DEGs. Using Spearman correlation analysis, the relationships between the stromal, stemness, and immune scores and the risk score were examined. In vitro experiments were all carried out independently in triplicate. For all, a *P*-value <0.05 was considered statistically significant.

## Results

### Differentially Expressed Angiogenesis and NETs-related Prognostic Genes

**Figure 2:**
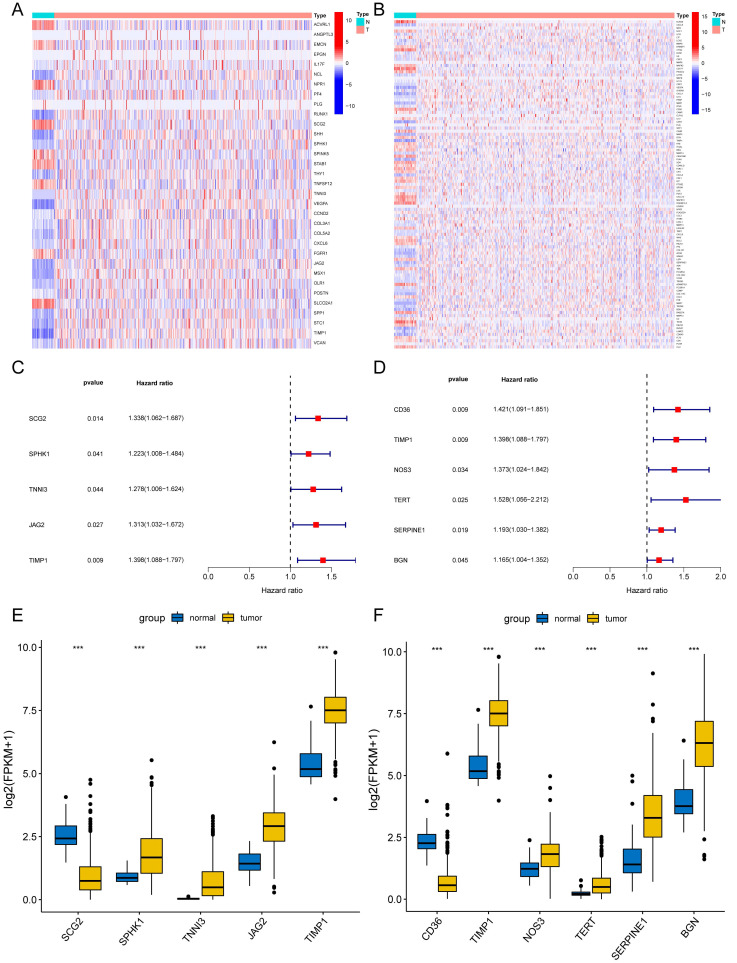
Differentially expressed angiogenesis-related and NETs-related genes with prognosis. Heatmaps of 33 differentially expressed angiogenesis-related genes (A) and 105 NETs-related genes (B) between tumor and normal tissues (*P*<0.05). Forest plots of five prognostic angiogenesis-related genes (C) and six prognostic NETs-related genes (D) by univariate Cox analysis (*P* < 0.05, coef > 0). The differential expression box plot of five prognostic angiogenesis-related genes (E) and six prognostic NETs-related genes (F) in COAD. *P < 0.05; **P < 0.01; ***P < 0.001.

We preliminarily screened 82 angiogenesis-related genes and 271 NETs-related genes through GSEA and GeneCards websites (correlation>10). The locations of 82 angiogenesis (Supplementary Fig. 1A) and 271 NETs-related genes (Supplementary Fig. 1B) were plotted by the package "Rcircos"^30^. In the TCGA-COAD set, 33 of 82 angiogenesis-related DEGs and 105 of 271 NETs-related DEGs were screened between COAD tissues (n = 480) and adjacent normal colon tissues (n = 41). DEGs were displayed in the heat map (Fig. 2A) (Fig. 2B). Through univariate Cox regression analysis, five angiogenesis-related DEGs (SCG2, SPHK1, TNNI3, JAG2, and TIMP1) and six NETs-related DEGs (CD36, TIMP1, NOS3, TERT, SERPINE1, and BGN) were identified with prognostic values (Fig. 2C) (Fig. 2D), serving as risk genes for the prognosis of COAD patients. TIMP1 was identified in both groups. The TCGA-COAD data showed higher expressions of SPHK1, TNNI3, JAG2, TIMP1, NOS3, TERT, SERPINE1, and BGN and lower expressions of SCG2, CD36 in COAD tissues than in the normal colon tissues (Fig. 2E) (Fig. 2F).

### Definition of API and NPI for COAD Patients

**Figure 3:**
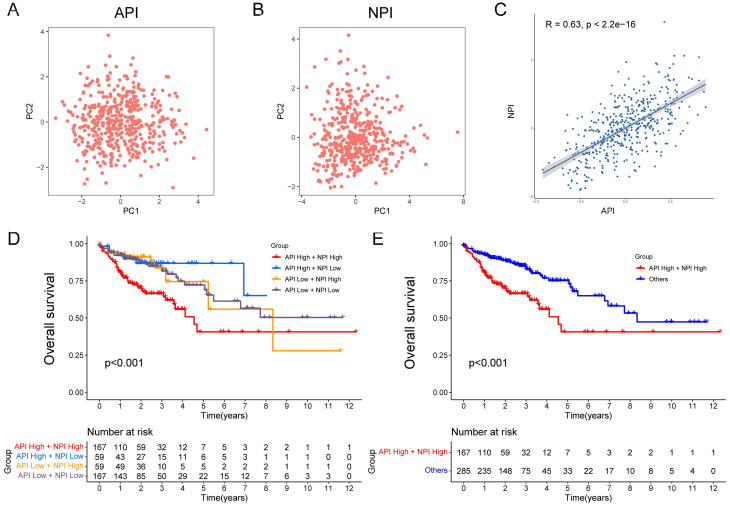
Construction of API and NPI with positive correlation. PCA analysis of five prognostic angiogenesis-related genes (A) and six prognostic NETs-related genes (B). (C) Scatter plot displaying the Spearman correlation of API and NPI. The KM plots displaying overall survival in four groups (D) and two newly defined groups (E).

The API and NPI were predicted according to the expression of the five angiogenesis-related DEGs (SCG2, SPHK1, TNNI3, JAG2, and TIMP1) and six NETs-related DEGs (CD36, TIMP1, NOS3, TERT, SERPINE1, and BGN) by PCA method (Fig. 3A-B). A strong correlation between API and NPI was identified (R=0.63, *P*<2.2e-16, Fig. 3C). COAD patients were grouped into high and low API /NPI groups depending on their median values. Four groups were categorized as follows: high API + high NPI group; high API + low NPI group; low API + high NPI group; low API + low NPI group. As expected, the high API + high NPI group of COAD patients exhibited a prominent survival disadvantage compared with the other three groups, illustrating that angiogenesis and NETs both contributed to a poor prognosis (Fig. 3D-E). The high API and high NPI molecular features defined a poor prognostic subgroup of COAD patients, which was worth further exploration.

### Characteristics of Immune Infiltration and TME in API and NPI Groups

The COAD patients were split into two groups, one with high API and the other with low API, according to the median API. We analyzed the overall survival of patients with high and low API, and the survival curve (Supplementary Fig. 2A) illustrated that patients with high API had a noticeably worse prognosis. Next, we utilized the ESTIMATE package to analyze the immune and stromal scores in these two groups. The outcomes demonstrated that the stromal score (Supplementary Fig. 2B) and immune score (Supplementary Fig. 2C) were higher in the high API group, which meant that the tumor tissues with high API scores had more abundant immune and stromal cells. Then, we applied the ssGSEA algorithm to assess the abundance of immune cells and functions in high and low API groups. In COAD, we discovered that the scores of 12 different types of immune cells and 13 different types of immune functions were higher in the high API group. Additionally, we identified ten widely accepted immune checkpoints, and we discovered that the high API group had a considerably increased expression of all ten immune checkpoints. Finally, we examined three types of cells limiting T-cell infiltration in tumors, namely, cancer-associated fibroblasts (CAF), myeloid-derived suppressor cells (MDSCs), and tumor-associated macrophages (TAMs). It was revealed that the low API group had a higher abundance of MDSCs and M2-TAMs, while the high API group had a higher infiltration of CAF. Additionally, T cells in the high API group had significantly higher scores for dysfunction and exclusion than T cells in the low API group.

We then analyzed NPI according to the same method and get similar results. The high NPI group had a worse prognosis (Supplementary Fig. 3A), higher stromal score (Supplementary Fig. 3B), and higher immune score (Supplementary Fig. 3C). The abundance of 13 immune cells (Supplementary Fig. 3D), 13 immune functions (Supplementary Fig. 3E), and 10 kinds of immune checkpoints (Supplementary Fig. 3F) were higher in the high NPI group. Lastly, the outcomes of the TIDE analysis (Supplementary Fig. 3G) were similar to those of API.

### Functional Analysis of high API/NPI Related DEGs

**Figure 4:**
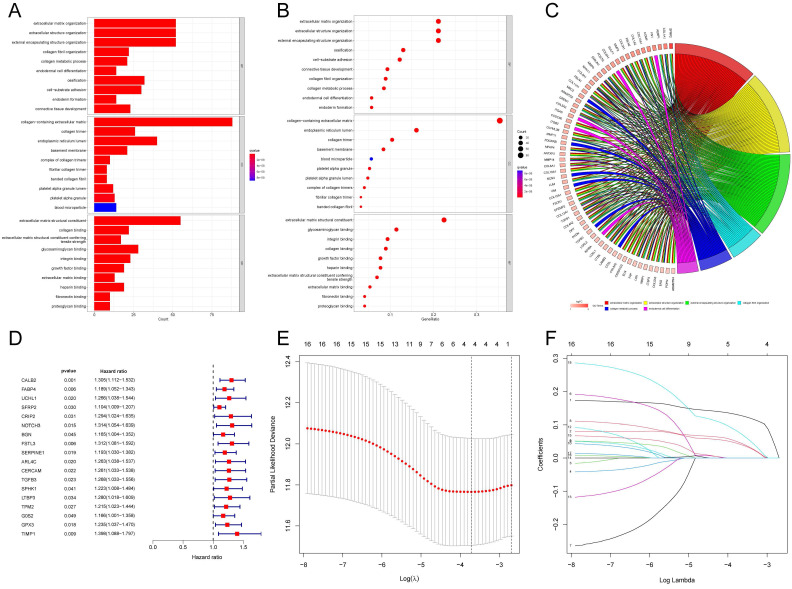
Gene Set Enrichment Analysis and Construction of the novel risk model. The results of GO enrichment analysis of the DEGs between two groups showing by barplot (A), bubble chart (B), and chord diagram (C). (D) Forest plot of 18 prognostic angiogenesis and NETs-related genes by univariate Cox analysis (*P* < 0.05). (E) 1000 cross-validation to determine the optimal penalty parameters lambda. (F) Lasso regression of the 18 prognostic angiogenesis and NETs-related genes.

According to the above results, API and NPI had a great correlation with prognosis and immune infiltration. The prognosis of the high API/NPI group was significantly different from the other three groups. Therefore, we wondered if it was practicable to build a prognosis model closely related to API and NPI at the same time. Then we analyzed the DEGs (| Log2FC |>0.8, *P*<0.05) of the high API + high NPI group compared with the other groups. The other three groups were referred to as the control group, while the high API + high NPI group was designated as the subject group. In the TCGA-COAD set, 258 DEGs were found to differ between the two groups. Functional enrichment analysis of these DEGs was performed. The top 30 significant terms of GO analysis illustrated that DEGs were mainly associated with the extracellular matrix, suggesting that these DEGs might function as vital regulators in the extracellular matrix of COAD. The outcomes of GO enrichment analysis were displayed by barplot (Fig. 4A), bubble chart (Fig. 4B), and chord diagram (Fig. 4C). The top significant terms of KEGG analysis revealed that DEGs were primarily connected with the phagosome, focal adhesion, and PI3K-Akt signaling pathway. The barplot, bubble chart, and cluster circle diagram (Supplementary Fig. 4A-C) were utilized to display the outcomes of the KEGG enrichment analysis.

### Construction and Validation of Risk Model

**Figure 5:**
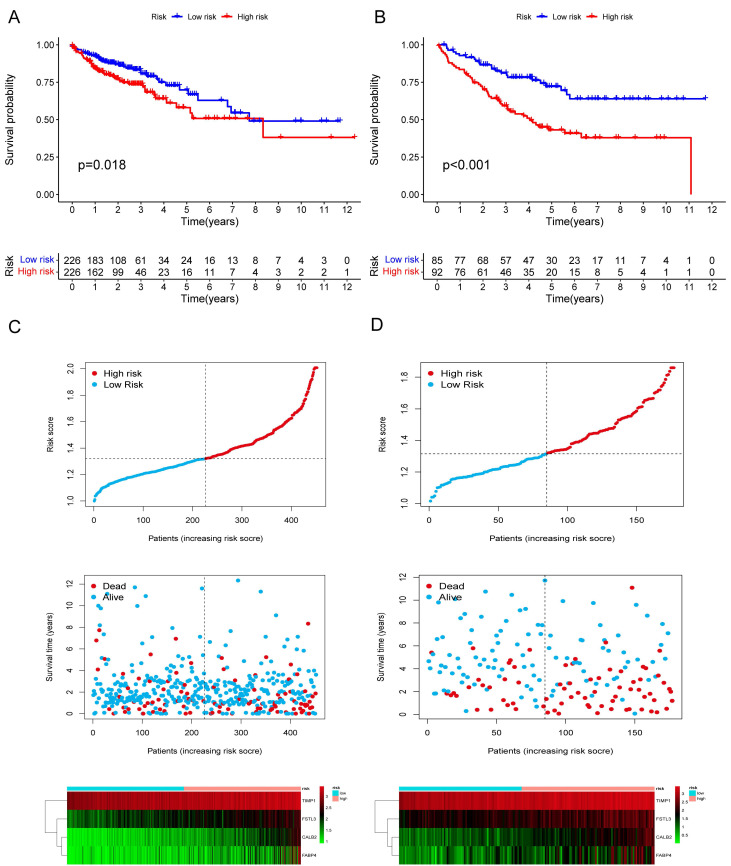
Validation of the prognostic risk model. The KM plots showing overall survival in train set (A) and test set (B). Scatter plots illustrating risk score distribution of the high-risk and low-risk groups and the relationship between survival time and risk score of the train set (C) and test set (D), as well as gene heat maps of these two sets.

**Figure 6:**
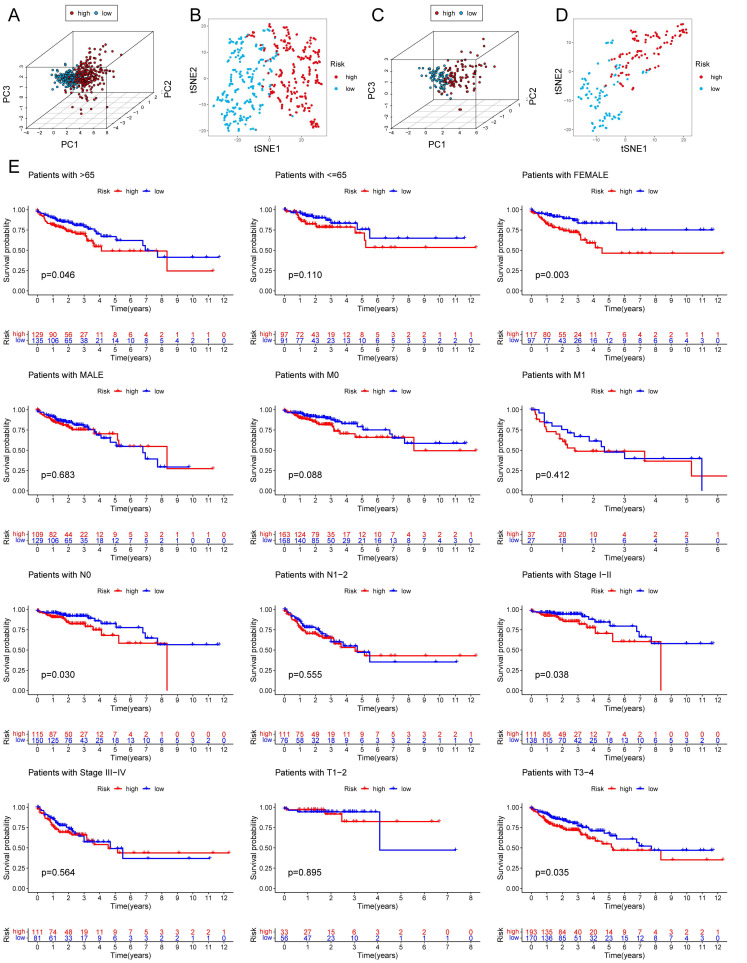
PCA analysis and survival analysis of clinicopathological features. The 3D scatter plots of PCA results of train set (A) and test set (C). The t-SNE analyses of train set (B) and test set (D). (E) KM plots in subgroups including age, gender, and tumor stages.

Among the 258 DEGs, 18 prognostic DEGs were identified (*P*<0.05). Using Cox regression analysis, the hazard ratio (HR) of 18 identified DEGs was determined (Fig. 4D). Through 1000 cross-validation, we determined the optimal penalty parameter λ (Fig. 4E) (Fig. 4F). Four genes (TIMP1, FSL3, CALB2, and FABP4) were selected in the risk score model with LASSO regression analysis. Risk score = TIMP1 × 0.0772652299695025 + FSL3 × 0.0472024839756706 + CALB2 × 0.128855340694661 + FABP4 × 0.0425233469315407. The median risk score was applied as the cutoff value when separating the COAD patients in the train set into the high-risk group (n = 226) and low-risk group (n = 226). A significantly better prognosis was found in the low-risk group than in the high-risk group (Fig. 5A). Based on the cut-off value of the train set, patients in the GSE17536 were also grouped into a high-risk group (n=92) and a low-risk group (n=85). Survival analysis of the test set (Fig. 5B) also revealed that a higher risk score was associated with a worse prognosis. The log-rank test was used to calculate the *P*-value for the train set (*P*=0.018) and test set (*P*<0.001). The test set's HR was 2.430 with a 95% confidence interval of 1.535 to 3.848, while the train set's HR was 1.612 with a 95% confidence interval of 1.084 to 2.398. The TCGA-COAD dataset had AUCs of 0.632, 0.621, and 0.609 for predicting 1-, 3-, and 5-year survival. While the AUCs of the GSE17536 set were 0.626, 0.640, and 0.667. We also visualized the risk plot, survival state map, and gene heat map of the train set (Fig. 5C) and the test set (Fig. 5D) respectively. The PCA and t-SNE plots indicated that subjects in these two groups occupied relatively different dimensions both in the TCGA (Fig. 6A-B) and the GEO set (Fig. 6C-D), suggesting that this model was able to differentiate patients in these two groups.

### Establishment of Nomogram

**Figure 7:**
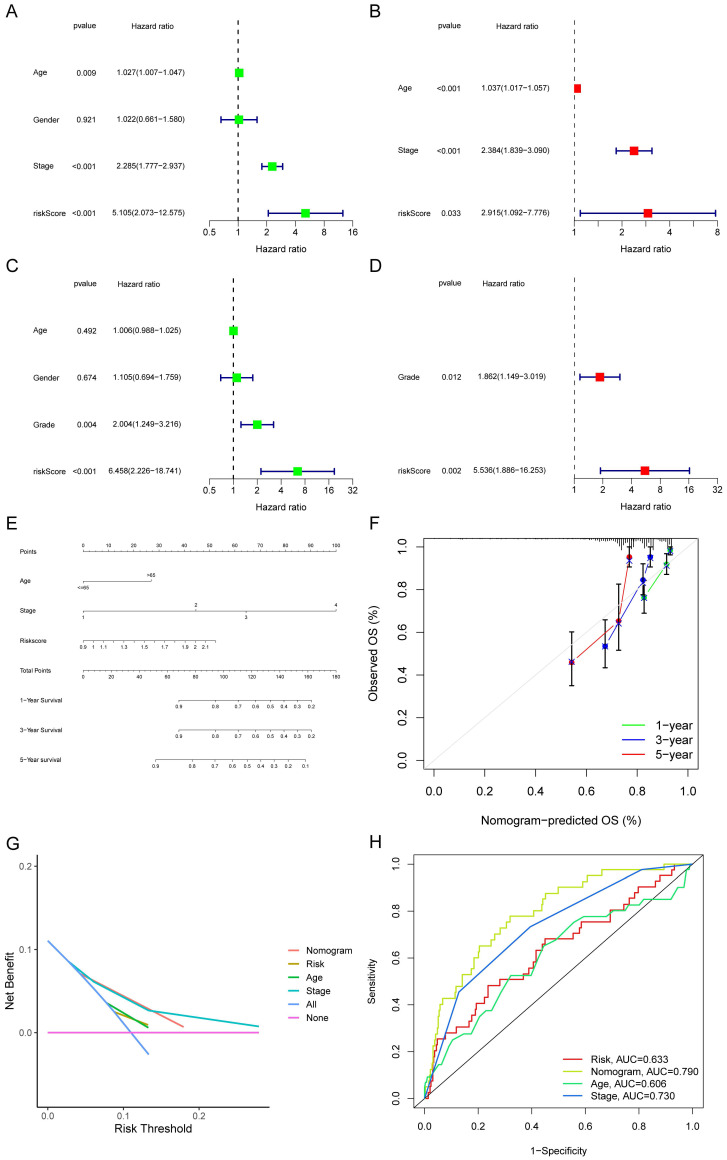
Forest plots and construction of a nomogram. Univariate Cox regression analyses displaying the association between the overall survival of patients and clinicopathological parameters along with the risk score in train set (A) and test set (C). Multivariate Cox regression analyses revealing independent prognostic factors in train set (B) and test set (D). (E) Nomogram depending on the risk score and other clinicopathologic features predicting the 1 -,-3 - and 5-year overall survival for COAD patients. (F) Calibration curves uncovering the consistency between predicted and observed 1-, 3- and 5-year overall survival rates in COAD patients based on the nomogram. DCA curve (G) and ROC curve (H) of nomogram, risk and other clinicopathologic features in COAD.

The TCGA-COAD set was adopted to evaluate the connection between the risk score and other clinical indicators (age, gender, TNM stage, and histological grade). Factors other than gender were all found to be strongly related to a bad prognosis through a univariate Cox analysis of overall survival (Fig. 7A). Age, stage, and risk score each contributed independently to a bad prognosis according to the multivariate analyses (Fig. 7B). In the GSE17536, the risk score was as well validated as an independent predictor of poor survival (Fig. 7C-D). A nomogram was formed utilizing meaningful indicators of univariate Cox analysis (Fig. 7E). The calibration curves were graphed (Fig. 7F). We also performed decision curve analysis (DCA) (Fig. 7G) and found that the curve of the nomogram was the farthest from the extreme curve. As a result, we concluded that the nomogram was more accurate in predicting survival. In the train set, the AUC of the nomogram was considerably larger than other features (Fig. 7H).

### Correlation with Immune Microenvironment

**Figure 8:**
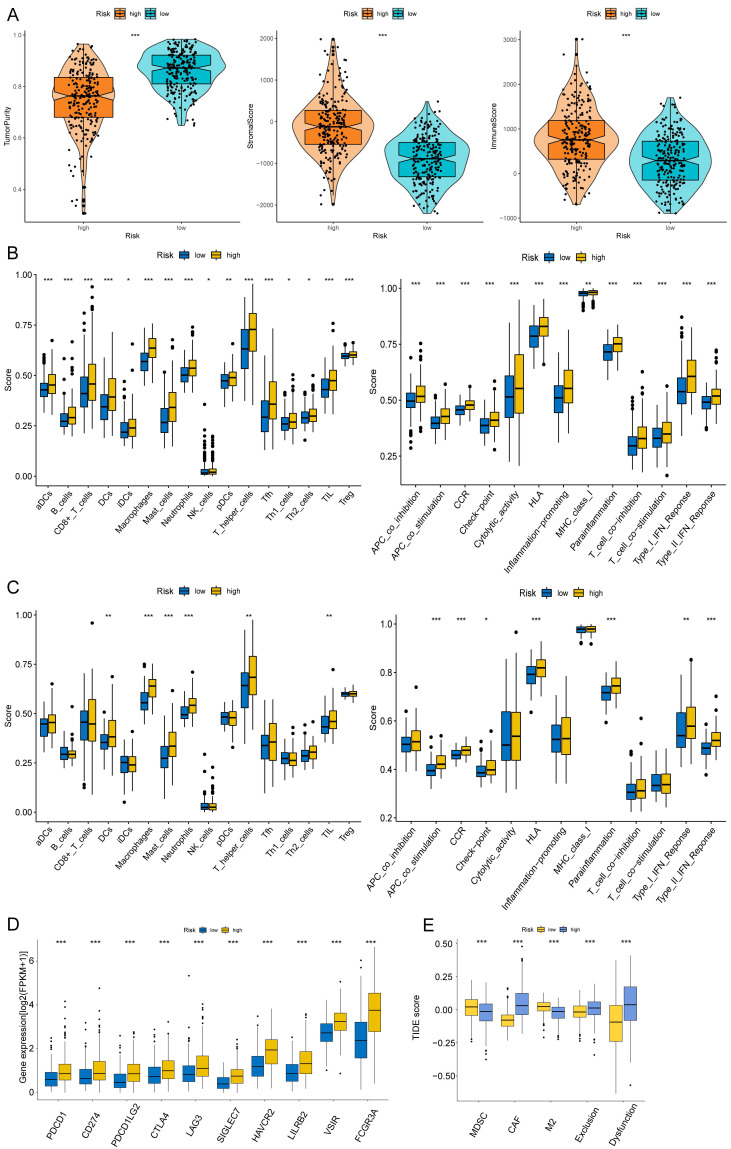
Immune infiltration analysis of the prognostic risk model. (A) The box-violin plots displaying the difference of the tumor purity, stromal score and immune score in two groups. The infiltrating levels of 16 immune cell types and the infiltrating levels of 13 immune functions in train set (B) and in test set (C) estimated by ssGSEA. (D) The expression box plots of 10 common immune checkpoints between low- and high-risk groups. (E) TIDE analysis revealing the difference of tumor immune dysfunction and exclusion in two groups. *P < 0.05; **P < 0.01; ***P < 0.001.

**Figure 9:**
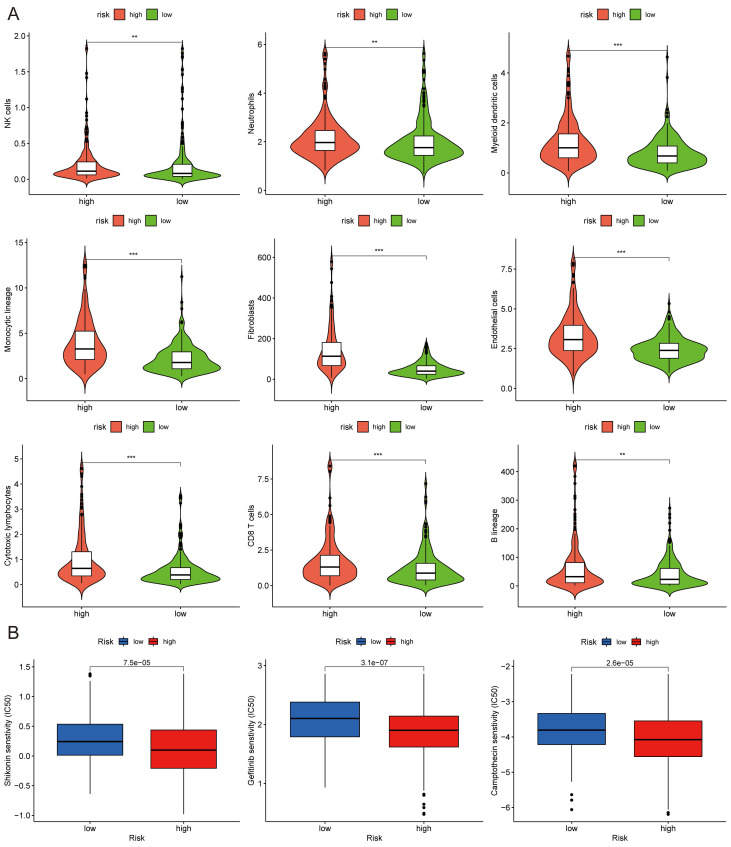
MCPcounter and drug sensitivity analyses of low-risk and high-risk group. (A) The violin diagrams showing the abundance of nine types of differentially expressed immune and stromal cells between two groups using MCPcounter. (B) Sensitivity difference of three common chemotherapeutic drugs in two groups. *P < 0.05; **P < 0.01; ***P < 0.001.

The correlations between the risk score and tumor immune microenvironment were investigated in the train set. The immune score, stromal score, ESTIMATE score, and tumor purity between the high and low-risk score groups were analyzed. The risk score was highly associated with the immune traits in the COAD patients. Significant differences were found concerning tumor purity, stromal score, and immune score between the two groups (Fig. 8A). The high-risk score was related to decreased tumor purity, increased stroma score, and increased immune score. Then, we utilized the ssGSEA package, finding that there were 16 immune cells and 13 immune functions upregulated in the high-risk group in the train set (Fig. 8B). In the test set, the high-risk group had six immune cells and seven immune functions upregulated compared with the low-risk group (Fig. 8C). Ten common immune checkpoints were analyzed, finding that all these immune checkpoints were considerably upregulated in the high-risk group (Fig. 8D). Finally, we applied TIDE tools to calculate tumor immune dysfunction and exclusion (Fig. 8E). We discovered that the CAF was higher in the high-risk group, while the abundance of MDSCs and M2 macrophages was higher in the low-risk group. Furthermore, the dysfunction and exclusion scores in the high-risk group were considerably higher. Moreover, the risk score and the quantity of some immune cells in the tumor microenvironment were highly associated. The abundance of nine types of cells in the tumor microenvironment were significantly higher in the high-risk group (Fig. 9A). Lastly, we found that the abundance of 24 MHC molecules was significantly higher in the high-risk group than in the low-risk group (Supplementary Fig. 5).

### Drug Sensitivity, TMB and Microsatellite Instability (MSI) Analysis

**Figure 10:**
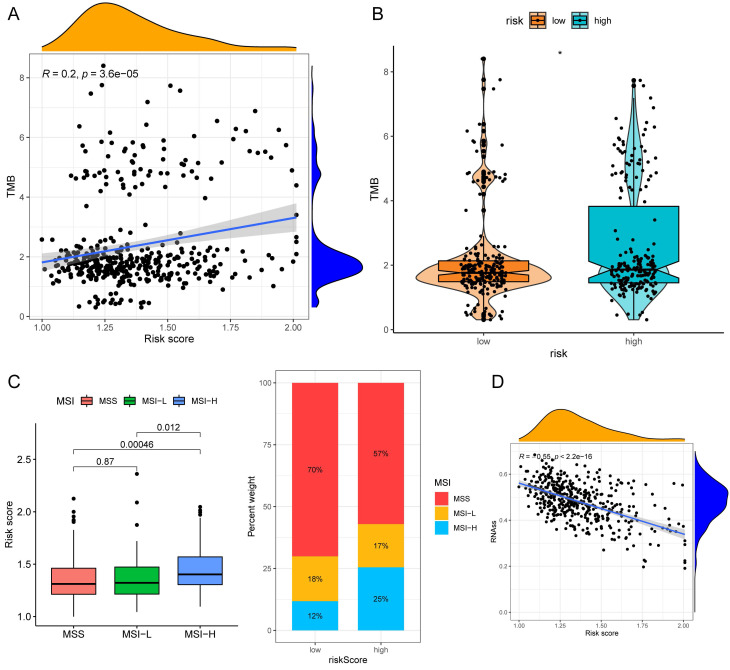
TMB and MSI analysis of the low- and high-risk groups. (A)Correlation scatter graph of TMB and Risk score. (B) The box-violin plot displaying the difference of the TMB score in two groups. (C) The risk scores in patients with different MSI statuses and the percentages of different MSI statuses in different risk score groups. (D) The correlation scatter plot between risk score and RNAss. *P < 0.05; **P < 0.01; ***P < 0.001.

The half-maximal inhibitory concentration (IC50) values of shikonin, gefitinib, and camptothecin were higher in the low-risk group (Fig. 9C), revealing that patients in the high-risk group might be more sensitive to these three medicines. The "maftools" package depicted the mutation status^31^. We plotted the waterfall maps of the top 20 genes with the highest mutation degree in the low-risk (Supplementary Fig. 6A) and high-risk groups (Supplementary Fig. 6B). The relationship between the risk score and TMB was proved to be modest but significant (Fig. 10A). The TMB score was higher in the high-risk group (Fig. 10B). In comparison to the MSS and MSI-L groups, the risk score in the MSI-H group was substantially higher (Fig. 10C). The risk score and tumor stem cells had a strong negative correlation (Fig. 10D).

### Experimental Verification of CALB2 in Vitro

**Figure 11:**
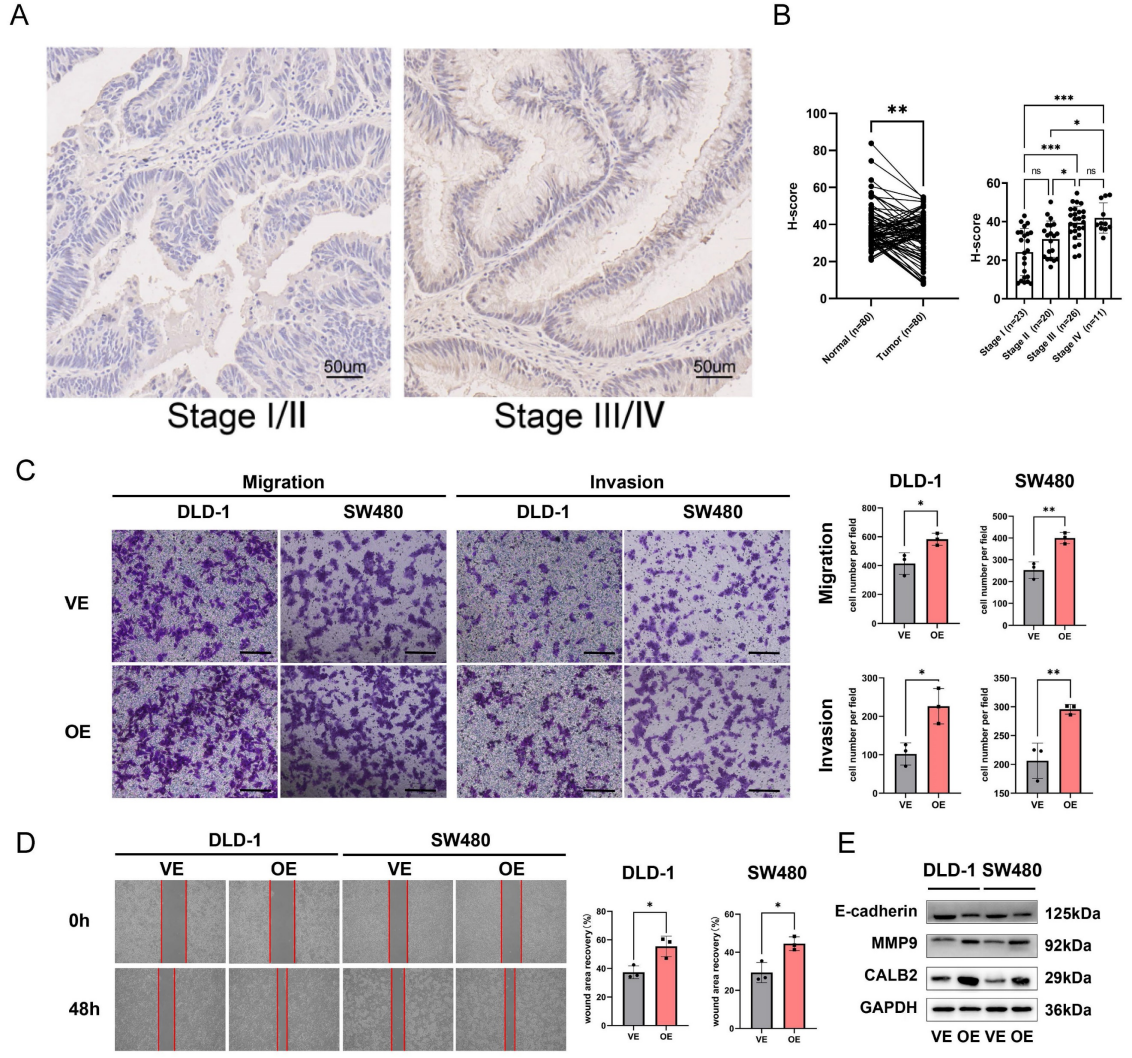
CALB2 promoted invasion and migration of CRC. (A) Representative immunohistochemical images of CALB2 in stage I/II and stage III/IV CRC tissues. Scale bar: 50um. (B) The expressions of CALB2 in stage III/IV CRC tissues were higher than those in stage I/II CRC tissues. (C) Transwell migration and invasion assays showed that CALB2 overexpression significantly promoted migration and invasion of DLD-1 and SW480 cells. The number of migratory and invasive cells were counted and plotted. (D) Wound-healing assay showed that CALB2 overexpression promoted wound area recovery. The wound area was measured and plotted. (E) CALB2 overexpression promoted upregulation of MMP9 and downregulation of E-cadherin as shown by western blot analysis.VE: Vehicle control; OE: Overexpression. * *P* < 0.05, ** *P* < 0.01, *** *P* < 0.001.

Among the model genes (TIMP1, FSL3, CALB2, and FABP4), the *P*-value of CLAB2 was 0.001 which was the lowest compared with those of the others and the role and function of CALB2 in CRC were rarely reported. So we tried to discover the function of CALB2 in CRC. The risk model described above was closely related to metastasis, we used the stage information of the COAD database and found that CALB2 was positively related to the tumor TNM stage (Supplementary Fig. 7A-B). Then we verified the expression of CALB2 protein in 80 paired normal and CRC tissues with IHC analysis (Supplementary Figure 8). CALB2 expression was significantly higher in stage III/IV CRC tissues than in stage I/II tissues, indicating a connection between CALB2 and tumor metastasis (Fig. 11B). Afterwards, we focused on how the clinicopathological features of the CRC patients related to the expression of CALB2 protein. We concluded that CALB2 protein level was significantly correlated with serum CEA level, tumor differentiation, lymphatic metastasis, distant metastasis, and TNM stage (Supplementary Table 3). Patients with high CALB2 showed considerably reduced disease-free survival and overall survival compared to those with low CALB2, according to the Kaplan-Meier analysis (Supplementary Fig. 7C). Then the function of CALB2 in CRC cells was analyzed in vitro. The overexpression of CALB2 was confirmed by WB after plasmid transfection. Transwell and wound-healing assays revealed that CALB2 overexpression significantly improved the migration and invasion of CRC cells (Fig. 11C-D). WB analysis showed that CALB2 overexpression promoted the upregulation of MMP9 and downregulation of E-cadherin in these CRC cells (Fig. 11E).

## Discussion

In this study, we focused on angiogenesis and NETs to interpret the invasion and metastasis of COAD. A novel prognostic model for COAD patients based on genes related to angiogenesis and NETs was developed with a stepwise approach. Firstly, differentially expressed and prognosis related angiogenesis and NETs-related genes were selected. A combination analysis of angiogenesis and NETs-related genes, reflecting the synergistic effects of angiogenesis and NETs, might have the potential to create a novel prognostic model of COAD. These prognostic DEGs were utilized to establish the API and NPI through the PCA method, respectively.

A subgroup of patients characterized by high API and high NPI were identified with an apparently poor survival. Secondly, the high API + high NPI group and the other three groups were defined. DEGs between these two groups were screened and functional analyses were conducted. GO and KEGG analysis revealed that those DEGs were correlated with extracellular matrix organization and cell-substrate adhesion, which was consistent with functions of NETs and angiogenesis and revealed the crosstalk between NETs and angiogenesis. 18 key DEGs were used as the targets of Lasso regression analysis and genes, including TIMP1, FSL3, CALB2, and FBAP4, were chosen to develop the risk score. The PCA and t-SNE analyses could differentiate between the high and low-risk patients based on their significantly different survival probabilities. Thirdly, a thorough exploration was performed to identify roles of the risk score with respect to clinicopathological characters, tumor immune infiltrates, et al. The multivariate Cox regression analysis suggested that the risk score was an independent prognostic factor. After the inclusion of clinical features, including age and stage, the nomogram established showed better prognosis predictability than the risk score alone, suggesting the importance of clinical features. To verify the predictive value of the risk model created, we applied it to the test set GSE17536 and obtained similar results. Increased mutation burden and immune system activity were characteristics of the high risk score, which meant an immune-inflamed tumor microenvironment. Many tumor infiltrating immune cells had higher levels in the high-risk group, which meant crosstalk between NETs and angiogenesis might promote an overall activation of immune function.

The exploration of key genes and their roles and functions that influence patient survival may promote the development of novel makers and treatment strategies for cancer patients. In this study, four key genes, TIMP1, FSL3, CALB2, and FABP4, were selected and included in constructing the prognostic model of COAD patients. All these genes were previously reported to play important roles in carcinogenesis. TIMP1 encodes a protein acting as a natural inhibitor of the matrix metalloproteinases (MMPs) and modulating tissue homeostasis. TIMP1 can promote tumor cell proliferation, inhibit apoptosis, influence angiogenesis, and facilitate tumor growth and metastasis^32^. According to an earlier study, aggressive tumor phenotypes were strongly associated with the abnormal expression of TIMP1 in COAD^33^. COAD tissues and metastatic lymph nodes possessed high levels of TIMP1 compared with normal colon tissues. For COAD patients, TIMP1 was an independent prognostic variable for both overall and disease-free survivals^34^. TIMP1 is a secretory protein that can be identified using the enzyme-linked immunosorbent assay (ELISA) in body fluids like blood, which makes it a candidate serum tumor marker^34^. High levels of systemic TIMP-1 were recognized as a bad prognostic factor^35^.

As a member of the follistatin-module protein family, follistatin-like 3 (FSTL3) is a secretory glycoprotein. FSTL3 in tumors was correlated with lymph node metastasis, clinical stage, tumor size, and intravascular emboli. FSTL3 was also discovered to be considerably expressed in COAD tissues compared to normal colon tissues. Patients with COAD who had increased FSTL3 expression would have a poor prognosis^36^. Increased FSTL3 expression promoted CRC cells to migrate and invade through β-catenin-mediated epithelial-mesenchymal transition (EMT) and aerobic glycolysis^37^. A recent bioinformatics study had identified FSTL3 as a key prognostic gene related to both immune status and lymph node metastasis in CRC^36^.

CALB2 is a subtype of the EF-hand family of Ca (2+)-binding protein. By altering the intrinsic apoptotic pathway, CALB2 controlled 5-fluorouracil sensitivity in colorectal carcinoma^38^. In COAD cells, butyrate may act as a negative regulator of CALB2^39^. It has been reported that CALB2 was significantly related to metastasis and prognosis in patients with hepatocellular carcinoma (HCC). HCC cell metastasis could be induced by CALB2 by activating the TRPV2-Ca2+-ERK1/2 signaling pathway^40^. However, there were few researches available regarding the role of CALB2 role in COAD. So we looked into how CALB2 influenced clinical outcomes in this study. Additionally investigated was the function of CALB2 on cell migration and invasion in vitro.

Fatty acid-binding protein 4 (FABP4), a fatty acid carrier protein, is involved in lipid transport, metabolism, and intracellular signal transduction^41^. According to a prior study using IHC, FABP4 protein expression was substantially higher in CRC tissues than in paracancerous tissues^42^. The differentiation, stage, and lymph node metastasis of tumors were all positively correlated with FABP4 protein expression in CRC tissues. When FABP4 was overexpressed, EMT was activated in COAD cells^43^,^44^.

The main cause of cancer-related deaths continues to be metastatic disease, which makes treating COAD extremely challenging. Angiogenesis and NETs were both thought to be important factors in metastasis and survival. The angiogenesis and NET-related genes were combined in the present research to construct a prognostic model for COAD. We believed that this study was the first to combine these gene signatures in order to predict overall survival with COAD patients. Four genes were chosen and included in the risk model for COAD prognosis centered on the investigation of the angiogenesis and NET-related genes. The risk score had shown a reliable predictive value in the GSE17536 validation set, and overall survival in the high-risk group was significantly lower than it was in the low-risk group. Compared to those of previous studies, we have developed an innovative prognostic model that differs from previously developed models, but its predictive value is not entirely the same. For example, in Xia et al.'s article^45^, the AUC value of the column line was only 0.7749, while our column line chart has a much higher predictive value than theirs. Furthermore, in Ping et al.'s article^46^, they constructed a prognostic model based on pyroptosis-related lncRNA, and the AUC value of the column line chart was 0.880, while our model has a relatively lower predictive value compared to theirs.

This study also had some limitations. Firstly, all the analyses were from public databases, and we needed more prospective studies to confirm our results. Second, the ethnic group of TCGA does not match that of our Chinese people. Using data of Caucasian to predict the prognosis of Chinese COAD patients would have genetic background differences. Thirdly, the numbers of evaluated NETs-representing genes and angiogenesis-representing genes were so small that may lack certain reliability. Fourthly, the average AUC value of the train set of the constructed prognosis model was less than 0.65, which was obviously inferior to other models in terms of accuracy. Moreover, although the AUC value of the nomogram was 0.790, there was a large difference between the calibration curve and the standard curve. Finally, the in vitro experiment of CALB2 proved that it could promote the invasion and metastasis of COAD cells. In vivo experiments might verify the findings.

## Conclusion

In summary, this study put forward new indexes (API and NPI) via the PCA method and established a novel four-gene prognostic risk score for COAD based on DEGs of the high API + high NPI group and the other groups. The risk score was independently associated with overall survival and some clinicopathological characteristics in COAD patients. Furthermore, we proved that the risk score was strongly correlated with immune infiltration and drug sensitivity. Finally, we selected CALB2 as a target for experimental verification and found that it could promote the invasion and migration of CRC cells in vitro.

## Supplementary Material

Supplementary figures and tables.

## Abbreviations

COAD: Colon adenocarcinoma
NETs: Neutrophil extracellular traps
API: Angiogenesis potential index
NPI: NETs potential index
DEGs: Differentially expressed genes
LASSO: Least absolute shrinkage and selection operator
TMB: Tumor mutation burden
TCGA: The cancer genome atlas
CNV: Copy number variation
GEO: Gene expression omnibus
PCA: Principal component analysis
GO: Gene ontology
KEGG: Kyoto encyclopedia of genes and genomes
ROC: Receiver operating characteristic
t-SNE: t-distributed stochastic neighbor embedding
TME: Tumor microenvironment
ESTIMATE: Estimation of STromal and Immune cells in MAlignant Tumours using Expression data
ssGSEA: single-sample gene set of the enrichment analysis
GSVA: Gene set variation analysis
HLA: human leukocyte antigen
MHC: Major histocompatibility complex
IC50: Half-maximal inhibitory concentration
MSI: Microsatellite instability
IHC: Immunohistochemistry
CRC: Colorectal cancer
WB: Western blotting
RIPA: Radio Immunoprecipitation Assay
BCA: Bicinchoninic acid
CAF: cancer-associated fibroblasts
MDSCs: Myeloid-derived suppressor cells
TAMs: tumor-associated macrophages
MMPs: Matrix metalloproteinases
TIMP1: TIMP Metallopeptidase Inhibitor 1
FSTL3: Follistatin-like 3
CALB2: Calbindin 2
FABP4: Fatty acid-binding protein 4
