# Supplementary material for: A Prognostic Model of Angiogenesis and Neutrophil Extracellular Traps Related Genes Manipulating Tumor Microenvironment in Colon Cancer
Source: J Cancer. 2023 Jul 9;14(11):2109–27. doi: 10.7150/jca.85778 (PMC10367930; doi:10.7150/jca.85778)
Supplement: Supplementary file 1 — Supplementary figures and tables. [file jcav14p2109s1.pdf]

Supplementary Material

Supplementary Figures

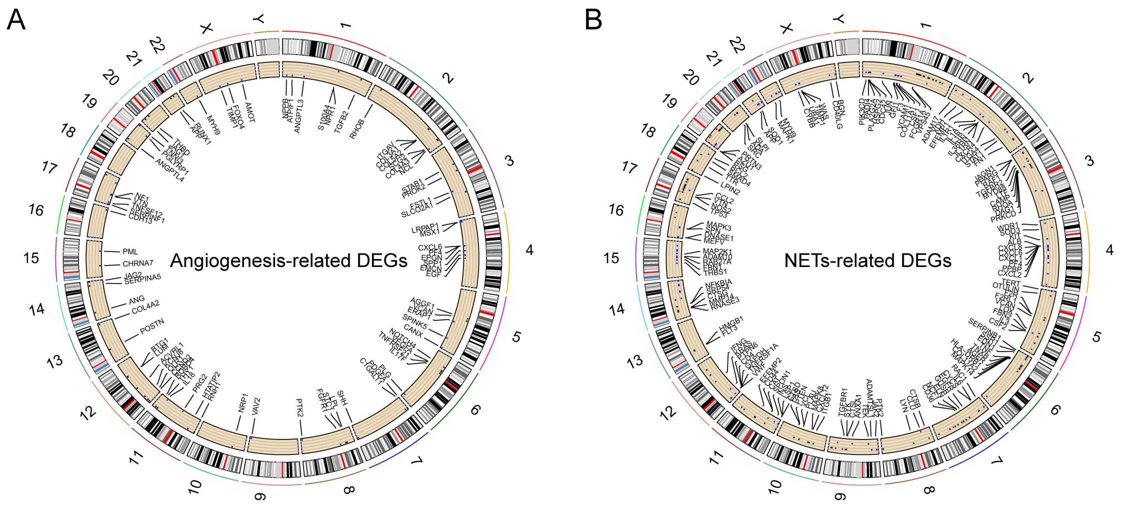

Supplementary Figure 1. CNV analysis of angiogenesis-related genes and NETs-related genes. (A)The location of angiogenesis-related genes on 23 chromosomes. (B)The location of NETs-related genes on 23 chromosomes.

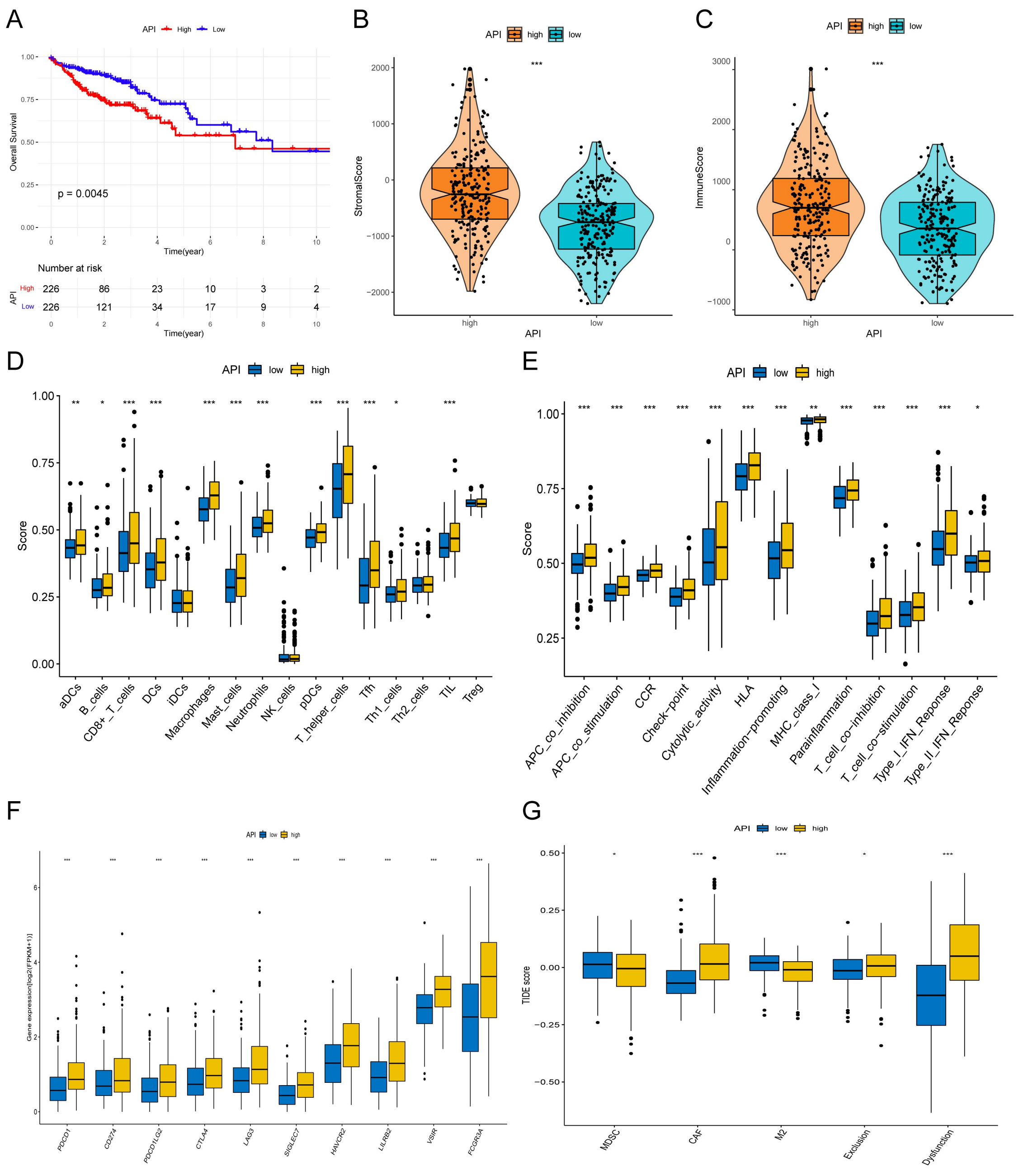

Supplementary Figure 2. Immune infiltration analysis of high API and low API groups. (A)The KM plot showing overall survival difference in high API and low API groups. The box-violin plot revealing the difference of the stromal score(B) and immune score(C) in high API and low API groups. The infiltrating levels of 16 immune cell types(D) and 13 immune functions(E) in high API and low API groups. (F)The expression box plot of 10 common immune checkpoints between high API and low API groups. (G)TIDE analysis revealing the difference of tumor immune dysfunction and exclusion in high API and low API groups. **P* < 0.05; ** *P* < 0.01; *** *P* < 0.001.

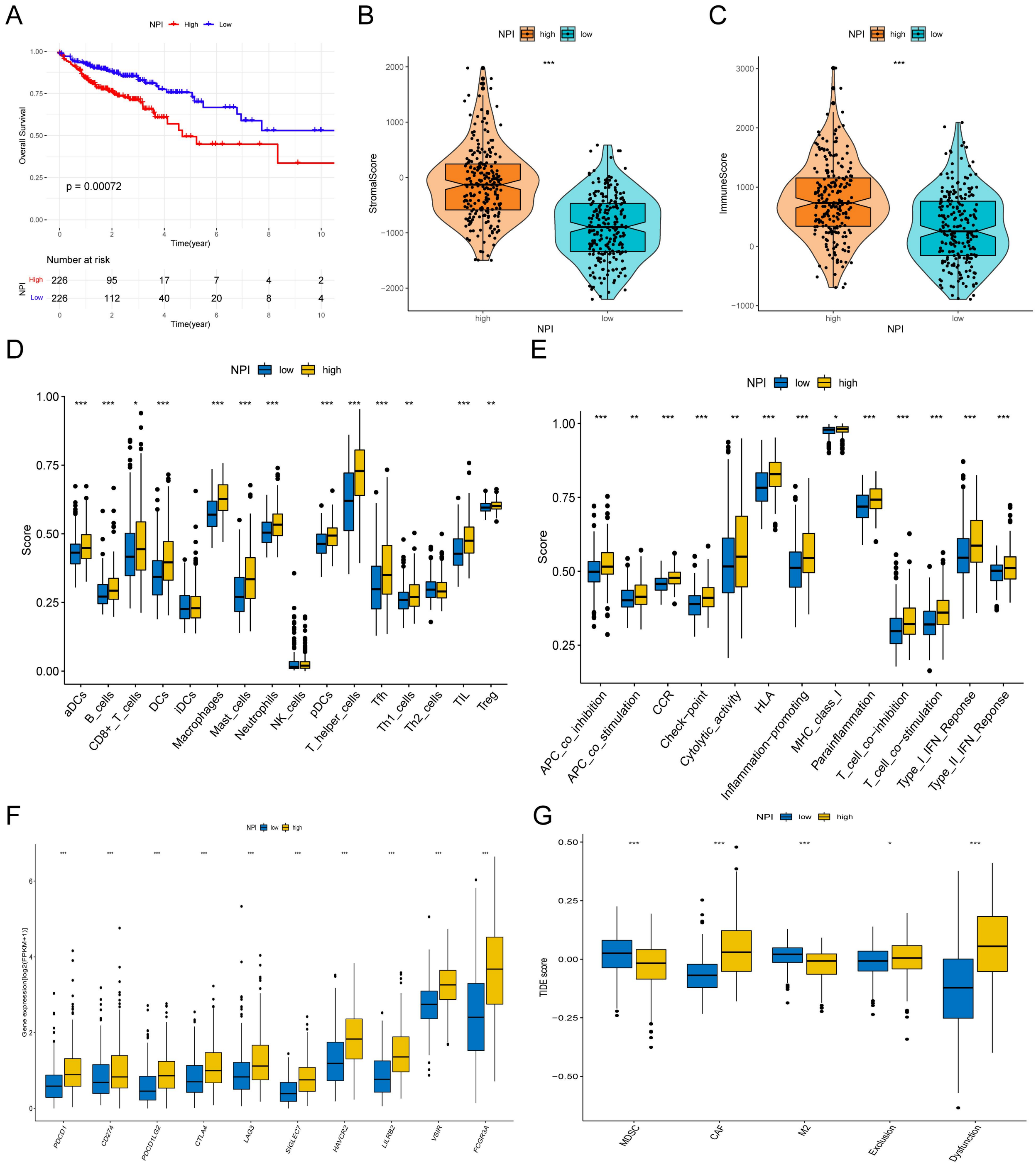

Supplementary Figure3. Immune infiltration analysis of high NPI and low NPI groups. (A)The KM plot showing overall survival difference in high NPI and low NPI groups. The box-violin plot revealing the difference of the stromal score(B) and immune score(C) in high NPI and low NPI groups. The infiltrating levels of 16 immune cell types(D) and 13 immune functions(E) in high NPI and low NPI groups. (F)The expression box plot of 10 common immune checkpoints between high NPI and low NPI groups. (G)TIDE analysis revealing the difference of tumor immune dysfunction and exclusion in high NPI and low NPI groups. * $P < 0.05$; ** $P < 0.01$; *** $P < 0.001$.

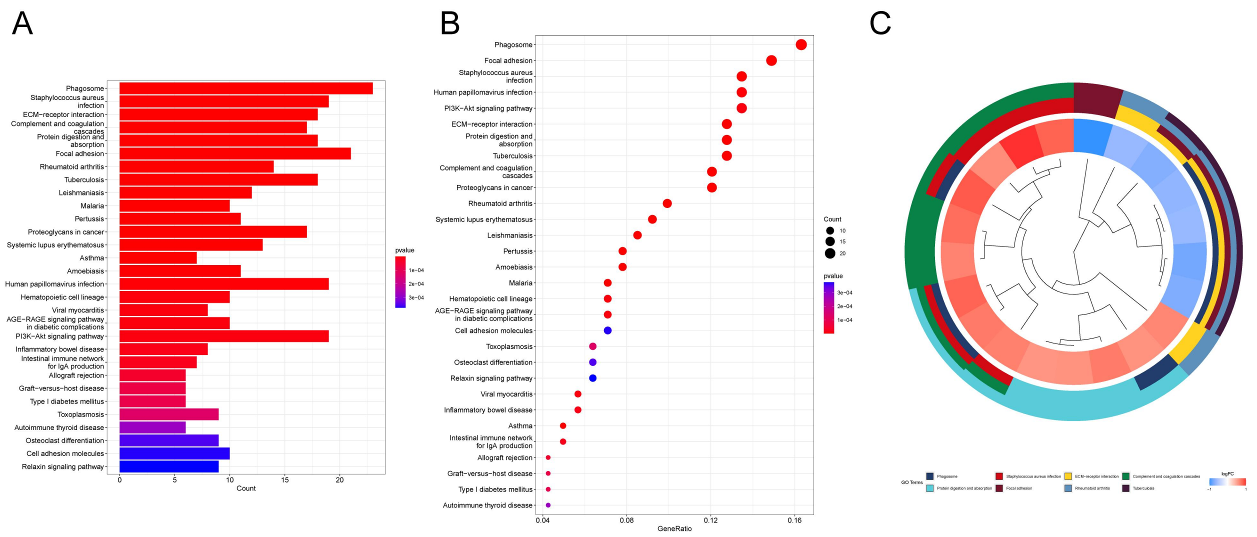

Supplementary Figure 4. The results of KEGG enrichment analysis of the differentially expressed genes showing by barplot (A), bubble chart(B), and cluster circle diagram(C).

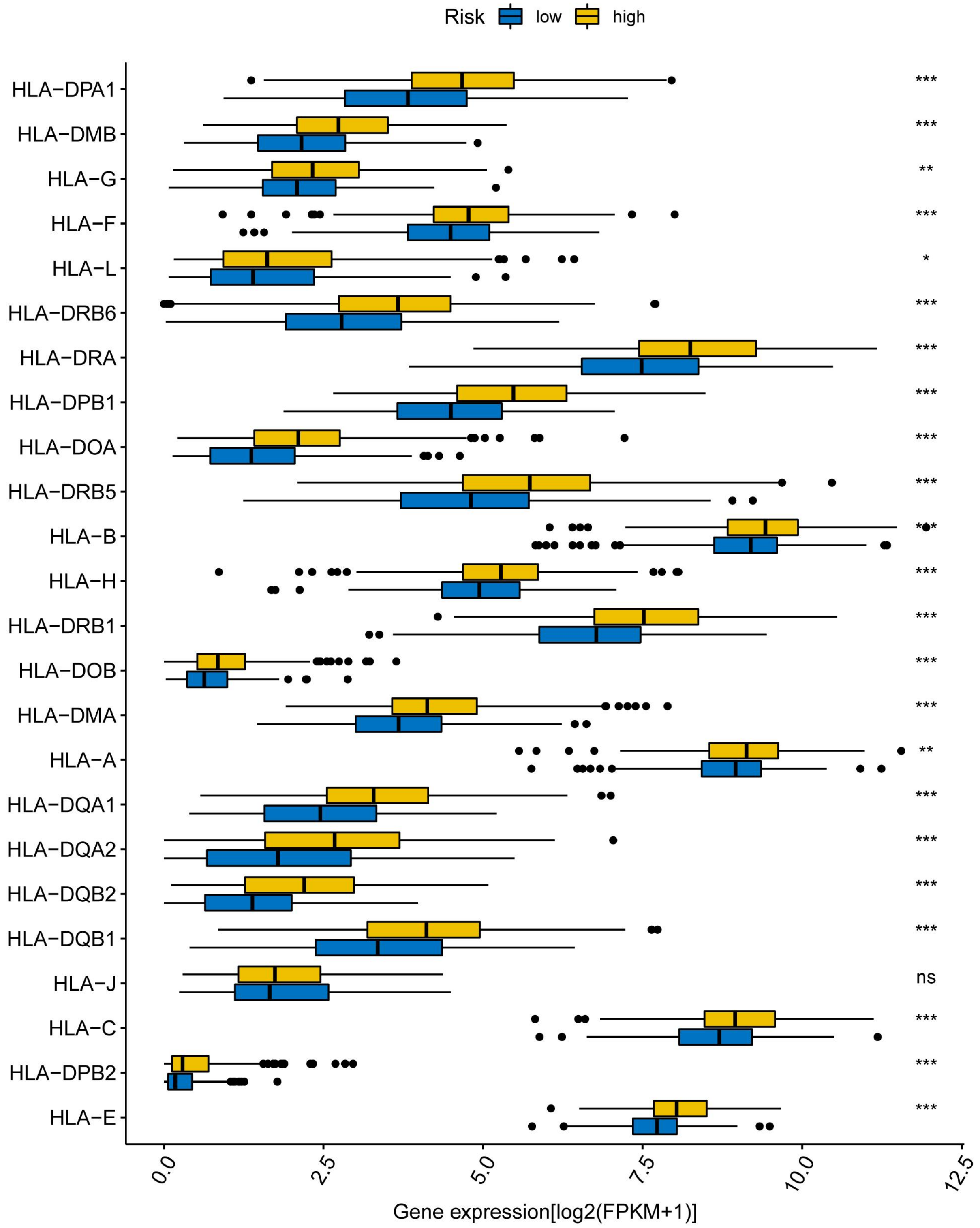

Supplementary Figure 5. Box plot of 24 MHC molecules expression level in two groups. * $P < 0.05$; ** $P < 0.01$; *** $P < 0.001$.

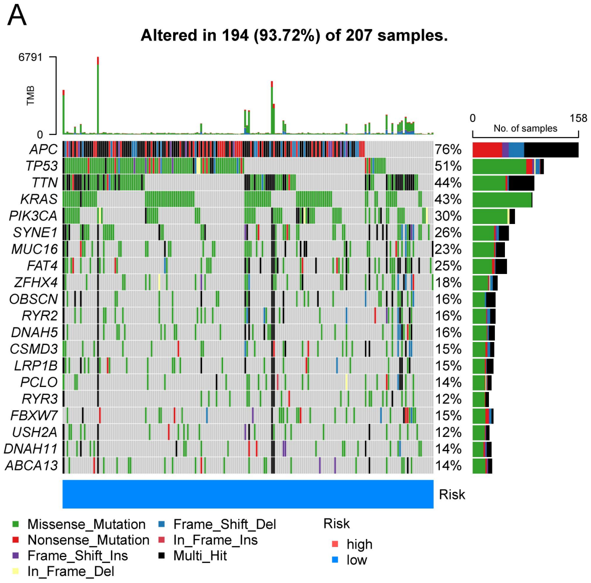

Supplementary Figure 6. Waterfall diagram of somatic mutation genes in the low- (A) and high-risk (B) groups.

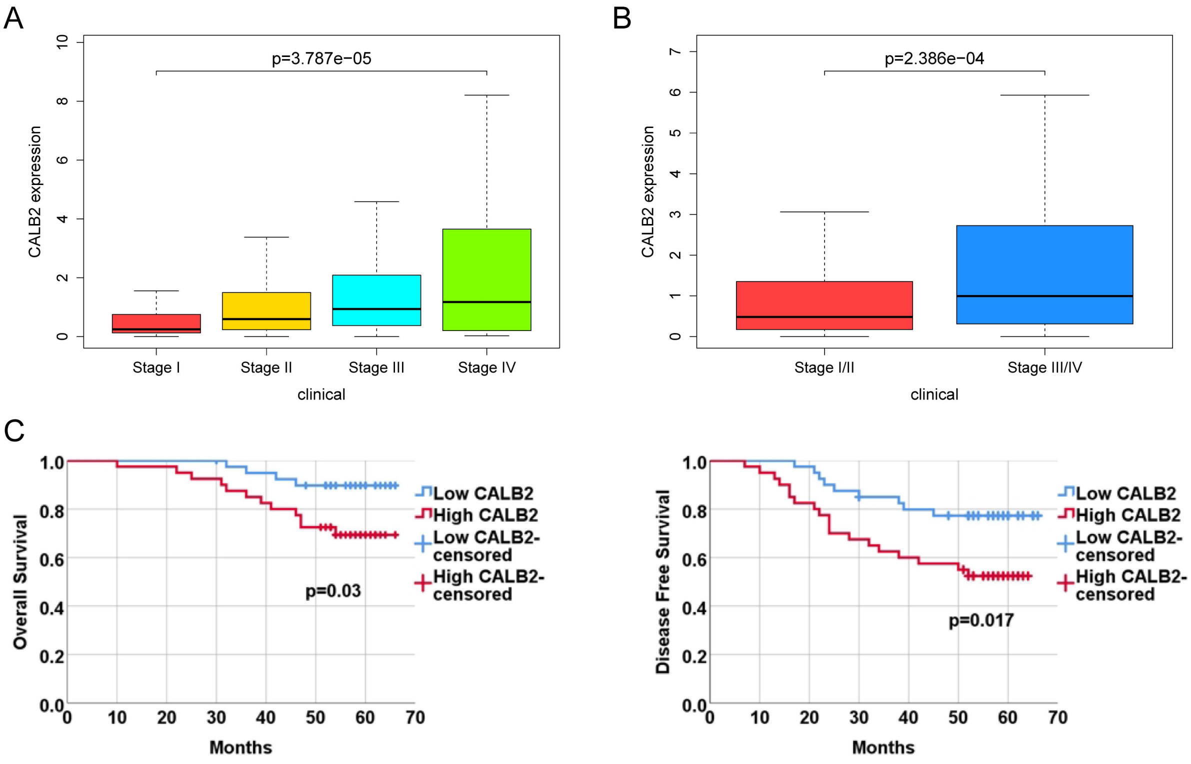

Supplementary Figure 7. The correlation of CLAB2 with clinical stage and survival. (A) CLAB2 expression level in Stage I, II, III, and IV tumors. (B) CLAB2 expression level in Stage I/II, III/IV tumors. (C)KM analysis showed the overall survival and disease-free survival in patients with high and low CALB2.

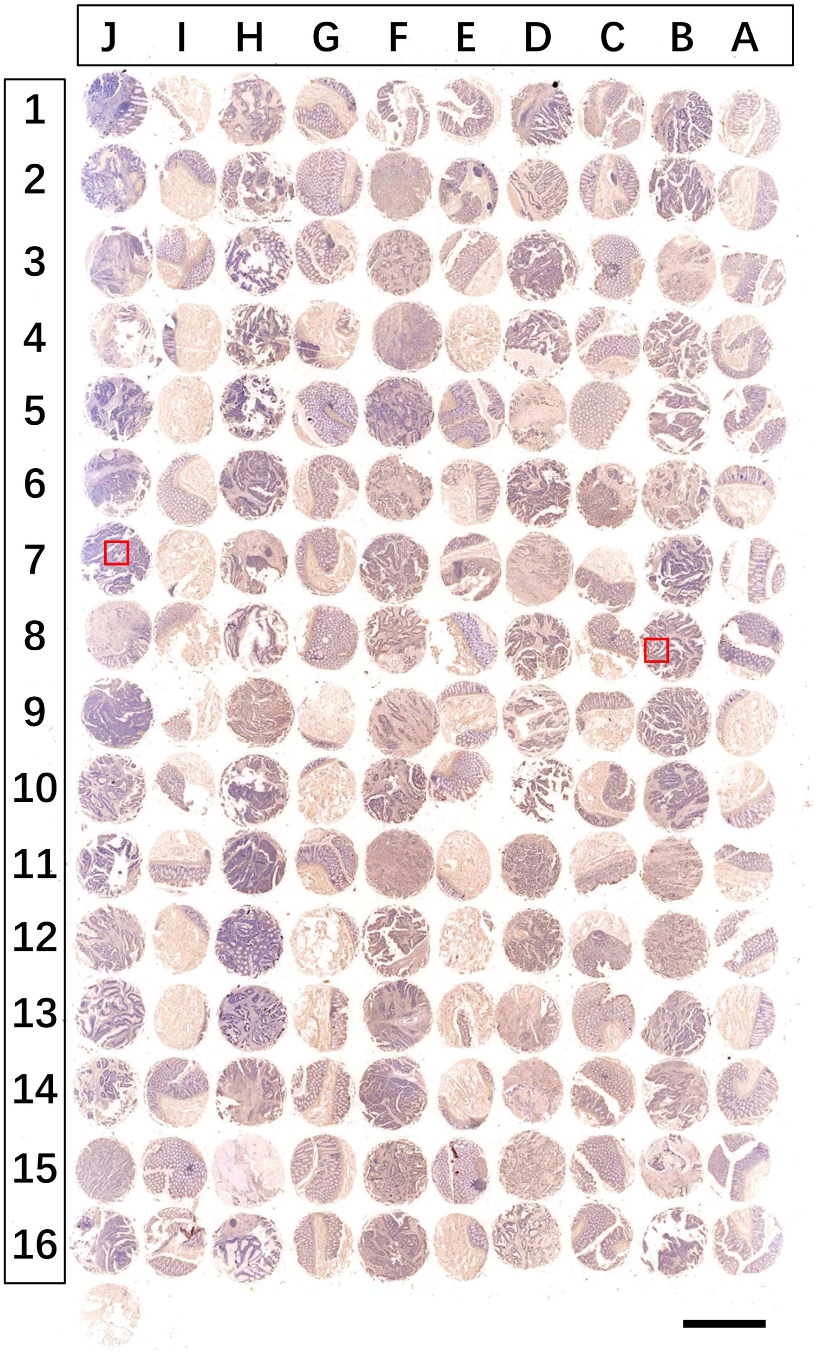

Supplementary Figure 8. The IHC staining of CALB2 in 80 paired normal and CRC tissues(bar：2000μm). Pictures in the red boxes are presented in Fig 11.

Supplementary Table1: Angiogenesis and NET -related genes

Angiogenesis and NET -related genes included in this study

Angiogenesis-related genes

ACVRL1	AGGF1	AMOT	ANG	ANGPTL3	ANGPTL4	APOH	APP	ATPIF1	BTG1
C1GALT1	CANX	CCND2	CDH13	CHRNA7	COL3A1	COL4A2	COL4A3	COL5A2	CXCL6
EGF	EMCN	EPGN	ERAP1	FGFR1	FOXO4	FSTL1	HTATIP2	IL17F	IL18
IL8	ITGAV	JAG1	JAG2	KCNJ8	LPL	LRPAP1	LUM	MSX1	MYH9
NCL	NF1	NOTCH4	NPPB	NPR1	NRP1	OLR1	PDGFA	PF4	PGLYRP1
PLG	PML	POSTN	PRG2	PROK2	PTK2	RHOB	RNH1	ROBO4	RUNX1
S100A4	SCG2	SERPINA5	SERPINF1	SHH	SLCO2A1	SPHK1	SPINK5	SPP1	STAB1
STC1	TGFB2	THBD	THY1	TIMP1	TNFRSF21	TNFSF12	TNNI3	VAV2	VCAN
VEGFA	VTN								

NET-related genes

ABL1	ACAN	ADA	ADA2	ADAM10	ADAM17	ADAMTSL1	AGER	AKT1	ALB
ANXA1	APOE	APP	AZU1	BCL2	BGN	BSG	C3	C5AR1	CALR
CAMP	CASP3	CASR	CAT	CCL2	CCL3	CCL5	CD177	CD36	CD4
CD40	CD40LG	CD44	CDC42	CDH1	CDKN3	CEACAM1	CEACAM8	CFH	CFTR
CLU	COL11A1	COL11A2	COL18A1	COL1A1	COMP	CRP	CSF1	CSF2	CSF3
CTSB	CTSD	CTSG	CTSL	CXCL1	CXCL12	CXCL2	CXCL6	CXCL8	CXCR1
CXCR2	CXCR4	CYBA	CYBB	CYCS	DAG1	DCN	DEFA1	DNASE1	DNASE1L3
ECM1	EDN1	EFEMP1	EFEMP2	EGF	EGFR	ELANE	ELFN2	ELN	ERBB2
F2R	F2RL1	F3	FAS	FASLG	FBLN1	FBLN5	FBN1	FBN2	FCAR
FCGR1A	FCGR2A	FCGR3A	FCGR3B	FGF2	FLT3	FN1	FOS	FPR1	FPR2
FRAS1	G6PC3	GAPDH	GATA2	GFI1	GUSB	HAX1	HLA-DRB1	HMCN1	HMGB1
HPSE	HSPG2	ICAM1	IFNG	IL10	IL17A	IL18	IL1A	IL1B	IL1R1
IL1RN	IL2	IL3	IL36RN	IL4	IL6	INS	ITGA4	ITGAL	ITGAM
ITGB1	ITGB2	ITGB3	JAGN1	JAK2	JUN	KIT	KRAS	KRIT1	LAMC1
LAMC2	LBR	LCN2	LCP2	LGALS3	LMNA	LOX	LOXL1	LPIN2	LSP1
LTF	LYN	MAP2K1	MAPK1	MAPK10	MAPK14	MAPK3	MAPK8	MEFV	MEPE
MIR223	MMP1	MMP12	MMP13	MMP14	MMP2	MMP3	MMP7	MMP8	MMP9
MPO	MRTFA	MYD88	MYH9	NCF1	NCF2	NCF4	NFKB1	NFKBIA	NLRP3
NOS2	NOS3	OTULIN	PADI4	PECAM1	PF4	PIK3CD	PIK3CG	PLA2G2A	PLA2G4A
PLAU	PLAUR	PLG	PPARG	PPBP	PRKCA	PRKCD	PRTN3	PSEN1	PSMB4
PSTPIP1	PTEN	PTGS2	PTK2	PTPN6	PTPRC	RAB27A	RAC1	RAC2	RAF1
RHOA	RNASE3	RUNX1	S100A8	S100A9	SELE	SELL	SELP	SELPLG	SERPINA1
SERPINA3	SERPINB1	SERPINE1	SIGLEC5	SLPI	SMAD3	SMAD4	SOD1	SOD3	SPARC
SPN	SPON1	SPP1	SRC	SRP54	SSR1	STAT1	STAT3	SYK	TCIRG1
TEK	TERT	TET2	TGFB1	TGFB2	TGFB3	TGFBR1	TGFBR2	THBS1	TIMP1
TLR2	TLR4	TNC	TNF	TNFRSF1A	TNXB	TP53	TREM1	TRPM2	TTR
UBA1	VCAM1	VCAN	VCL	VEGFA	VPS45	VTN	VWF	WAS	WDR1
XDH									

Supplementary Table2: H-SCORE of CALB2 in 80 paired tissues

Rank	CALB2 H-score (normal)	CALB2 H-score (tumor)	Time to death (months)	Death	Rank	CALB2 H-score (normal)	CALB2 H-score (tumor)	Time to death (months)	Death
A1	60.6154	53.4591	31	Yes	E9	32.5066	49.4784	54	Yes
A2	48.8952	38.8956	58	No	E10	33.6733	32.6406	60	No
A3	38.5205	36.141	46	Yes	E11	28.4567	34.7623	60	No
A4	64.0265	41.3888	62	No	E12	28.7382	34.2686	60	No
A5	40.7074	39.0728	56	No	E13	28.2188	34.3567	59	No
A6	57.1279	35.1659	57	No	E14	25.5224	40.0209	59	No
A7	47.663	31.6984	64	No	E15	36.8296	44.9666	59	No
A8	32.0367	20.5722	57	No	E16	55.3927	43.0051	59	No
A9	45.723	36.512	51	No	G1	44.9099	24.5028	59	No
A10	45.0529	31.5002	65	No	G2	83.8591	53.8988	10	Yes
A11	32.007	43.7656	54	No	G3	34.3432	42.4479	57	No
A12	21.7664	37.2143	39	Yes	G4	38.9727	43.801	32	Yes
A13	34.2671	38.3555	54	No	G5	37.9821	30.0177	48	No
A14	20.905	36.5026	25	Yes	G6	54.9373	52.3878	47	Yes
A15	40.9922	35.2204	53	No	G7	38.6019	41.7435	55	No
A16	26.8108	38.2272	47	Yes	G8	32.0744	8.4701	54	No
C1	26.5515	33.8123	53	No	G9	36.5659	19.8006	30	loss
C2	34.991	33.738	53	No	G10	22.299	37.7541	55	No
C3	31.4849	7.8695	53	No	G11	48.289	25.0457	54	No
C4	50.6069	32.8966	53	No	G12	30.6784	43.3013	53	No
C5	74.3488	54.7962	58	No	G13	24.9976	37.7204	53	No
C6	33.1338	38.6207	61	No	G14	30.7551	38.0951	53	No
C7	43.1425	29.9264	52	No	G15	34.9868	41.57	41	Yes
C8	32.3626	39.2464	52	No	G16	43.8981	34.5512	52	No
C9	35.8251	33.099	36	Yes	I1	32.6395	16.4714	52	No
C10	25.4782	33.68	48	loss	I2	35.2171	18.2528	52	No
C11	56.8146	39.667	66	No	I3	34.9956	20.5237	56	No
C12	44.9525	30.4492	65	No	I4	33.1076	25.0449	66	No
C13	56.8245	28.0402	46	Yes	I5	33.4335	21.0183	66	No
C14	48.8237	46.532	64	No	I6	34.5234	20.6661	65	No
C15	33.9318	36.7738	36	Yes	I7	33.4624	22.371	65	No
C16	38.4466	45.1857	63	No	I8	36.8783	21.7513	42	Yes
E1	29.1786	32.7333	65	No	I9	42.3849	21.8181	57	No
E2	30.3727	38.7709	62	No	I10	29.6762	8.0056	63	No
E3	35.065	46.4582	22	Yes	I11	34.2215	11.4185	56	No
E4	35.8363	27.063	62	No	I12	39.1818	50.5295	62	No
E5	35.7223	50.2491	61	No	I13	30.5714	11.0216	62	No
E6	37.3736	43.8332	60	No	I14	30.1412	8.8961	58	No
E7	30.9272	23.7567	32	Yes	I15	37.8248	9.2151	52	No
E8	31.6736	50.184	60	No	I16	38.4083	14.2978	58	No

Supplementary Table3: The correlation of CALB2 expression and clinicalpathological parameters

	Low CALB2(n=40)	High CALB2(n=40)	*P*
Gender			0.356
Male	27	23	
Female	13	17	
Age(years)			0.262
<65	21	16	
≥65	19	24	
BMI (kg/m^2)			1
<24	27	27	
≥24	13	13	
CEA(μg/L)			0.022
<4.7	29	19	
≥4.7	11	21	
CA19-9(U/ml)			0.21
<39	36	32	
≥39	4	8	
Location			0.265
Right colon	11	10	
Left colon	6	12	
Rectum	23	18	
Differentiation			0.009
Low/Medium	32	21	
High	8	19	
Tumor diameter(cm)			0.478
<4	15	12	
≥4	25	28	
Lymphatic metastasis			<0.001
No	32	14	
Yes	8	26	
Distant metastasis			0.003
No	39	30	
Yes	1	10	
TNM stage			<0.001
I/II	32	11	
III/IV	8	29	
Lymphovascular invasion			0.189
No	33	28	
Yes	7	12	
Perineural invasion			0.239
No	35	31	
Yes	5	9	
